# Orientation-Based Control of Microfluidics

**DOI:** 10.1371/journal.pone.0149259

**Published:** 2016-03-07

**Authors:** Nazila Norouzi, Heran C. Bhakta, William H. Grover

**Affiliations:** Department of Bioengineering, University of California, Riverside, Riverside, CA, United States of America; Texas A&M University, UNITED STATES

## Abstract

Most microfluidic chips utilize off-chip hardware (syringe pumps, computer-controlled solenoid valves, pressure regulators, etc.) to control fluid flow on-chip. This expensive, bulky, and power-consuming hardware severely limits the utility of microfluidic instruments in resource-limited or point-of-care contexts, where the cost, size, and power consumption of the instrument must be limited. In this work, we present a technique for on-chip fluid control that requires no off-chip hardware. We accomplish this by using inert compounds to change the density of one fluid in the chip. If one fluid is made 2% more dense than a second fluid, when the fluids flow together under laminar flow the interface between the fluids quickly reorients to be orthogonal to Earth’s gravitational force. If the channel containing the fluids then splits into two channels, the amount of each fluid flowing into each channel is precisely determined by the angle of the channels relative to gravity. Thus, any fluid can be routed in any direction and mixed in any desired ratio on-chip simply by holding the chip at a certain angle. This approach allows for sophisticated control of on-chip fluids with no off-chip control hardware, significantly reducing the cost of microfluidic instruments in point-of-care or resource-limited settings.

## Introduction

The advantages of microfluidics over conventional lab-scale techniques—reduced reagent consumption, faster reactions, smaller instrument size, enhanced automation, higher throughput, and so on—have enabled applications for microfluidic instruments in fields as diverse as health care, environmental monitoring, and space exploration. The ability to control fluid flow on a microfluidic chip is a fundamental need in these instruments. However, most existing techniques for controlling on-chip fluid flow rely on off-chip hardware. For example, to control the mixing ratio of two fluids in a microfluidic chip, two off-chip pumps (pressure regulators or syringe pumps) are typically required. Valves and pumps can be moved on-chip [1, 2], but microfluidic valves and pumps also require off-chip hardware (usually computer-controlled solenoid valves and pressure or vacuum pumps). Electrical methods for controlling fluids, such as dielectrophoresis [3, 4] and electrowetting, [5] require off-chip electrical power supplies and complicate the fabrication of the microfluidic chip. In each of these approaches, the off-chip hardware required to control on-chip fluid flow can *cost thousands of dollars, consume hundreds of watts of electrical power, and contribute significant bulk to the instrument*. Consequently, many microfluidic instruments remain unsuitable for use in point-of-care or resource-limited settings, where instrument cost, power consumption, and size must be minimized.

**Fig 1:**
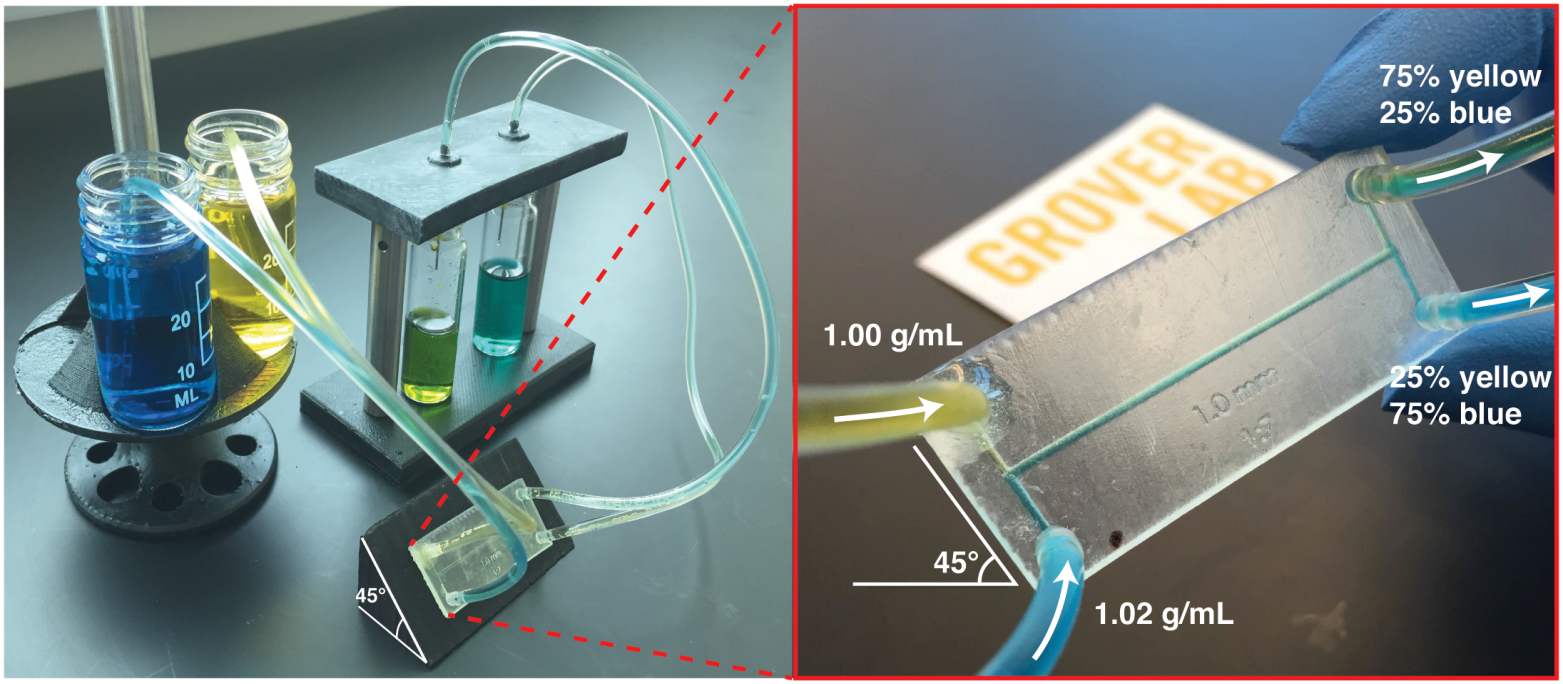
Using the orientation of a microfluidic chip to control the mixing ratio of fluids on-chip. Two fluids (yellow and blue) flow into the chip; the blue fluid includes an additive (sucrose) that makes the blue fluid 2% more dense than the yellow fluid. When the two fluids flow together in the chip, the fluids rotate to orient the more-dense blue fluid toward Earth’s gravity. When the channel then splits, the amount of each fluid flowing in each direction is precisely controlled by the angle of the chip. By using this approach, any desired mixing ratio of the yellow and blue fluids can be obtained simply by holding the chip at a certain angle; no off-chip control hardware is needed.

Here we describe a method for precisely controlling fluid flow inside a microfluidic chip that requires *no off chip hardware*. In this method, by simply orientating a microfluidic chip at a certain angle, on-chip fluids can be routed and mixed in any desired ratios. We accomplish this by adding inert compounds that slightly increase the density of certain fluids in the chip. When two fluids of different densities flow together under laminar flow on-chip, the interface between the two fluids quickly reorients itself to be orthogonal to Earth’s gravitational force. If the channel containing the fluids then splits into two channels, the amount of each fluid flowing into each channel is a function of the angle of the channels relative to gravity. In this manner, different amounts of different fluids can be routed on-chip simply by changing the orientation of the microfluidic chip as shown in Fig 1.

As a proof-of-concept demonstration, we used the principle of orientation-based microfluidic control in a simple mixer chip that is capable of generating any desired mixing ratio of two fluids. We chose to create a mixer because of the importance of mixing in microfluidic devices. Microfluidic mixers automate the time-consuming process of preparing arbitrary concentrations and mixtures of solutions by hand. Most existing microfluidic mixers utilize either microfluidic valves and pumps [1, 2] or arrays of split-and-combine operations [6, 7]. These mixers have found a variety of uses in microfluidic chips for evolving novel ribozymes [8], cytotoxicity studies [9], estimation of drug efficiency and optimization of biochemical reactions [10, 11]. However, a chip that uses these existing mixers is capable of generating only certain fixed ratios of mixtures; it cannot be used to generate *any* desired concentration or mixing ratio without redesigning the chip. Additionally, mixers containing microfluidic valves and pumps still rely on off-chip hardware for controlling these valves and pumps. Finally, existing valve- and pump-based or split-and-combine mixers consume a relatively large amount of fluid to generate a relatively small amount of the desired mixture. In summary, there is an unmet need for simple, equipment-free methods for generating arbitrary mixtures of fluids in microfluidic chips.

## Theory of orientation-based microfluidics

**Fig 2:**
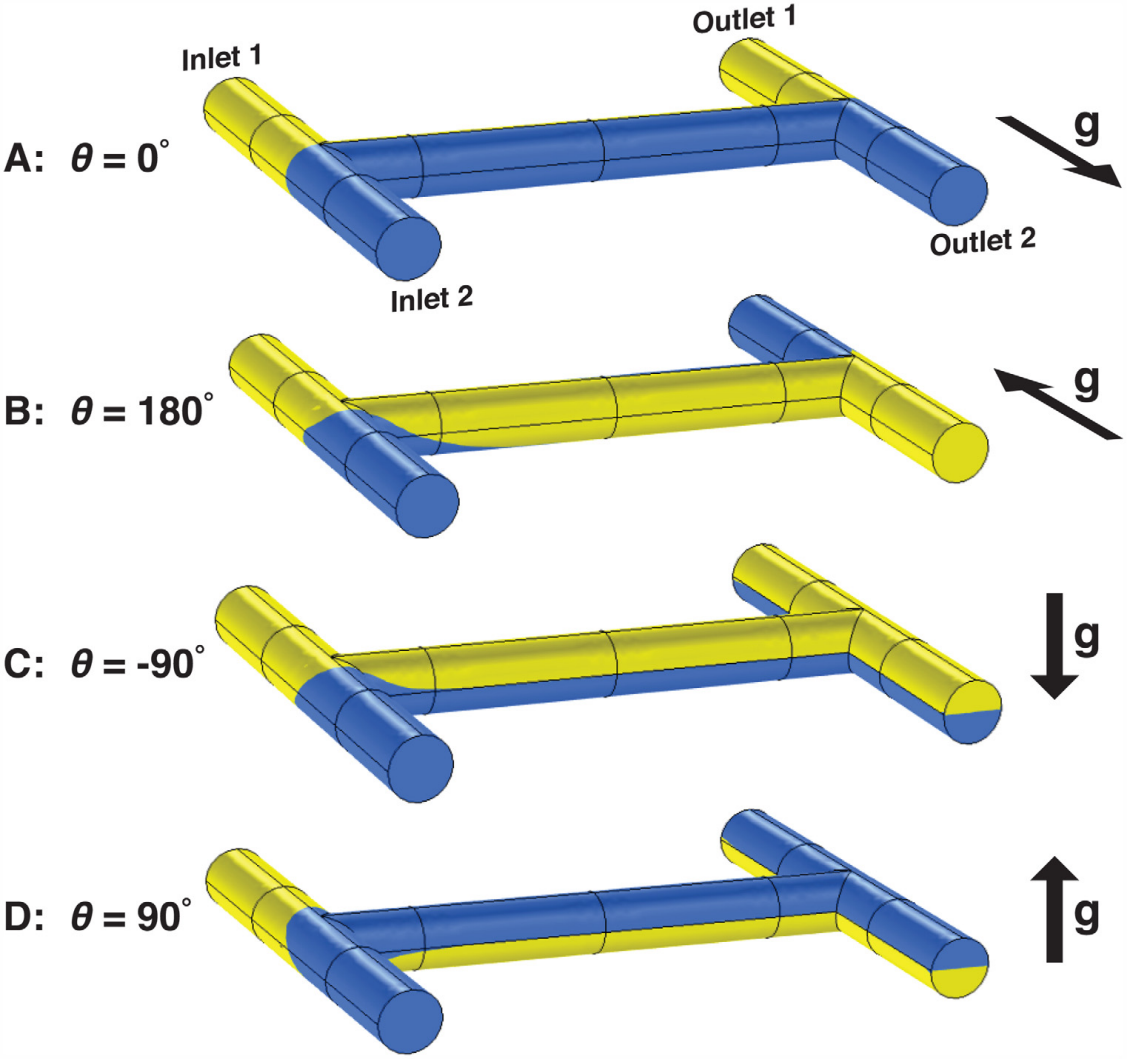
Principle of orientation-based control of microfluidics. These simulations show fluid flowing inside a simple microfluidic channel network containing two inlets and two outlets. Inlet 1 contains a less-dense yellow fluid and Inlet 2 contains a more-dense blue fluid. When the chip is oriented such that Inlet 2 is aligned with the Earth’s gravitational force **(A)**, the yellow and blue fluids remain unperturbed and exit the chip in the same directions from which they entered (yellow at Outlet 1 and blue at Outlet 2). However, when the chip is rotated 180° **(B)**, the force of gravity causes the fluids to swap places in the horizontal channel, placing the more-dense blue fluid on the bottom of the channel and the less-dense yellow fluid on the top. Consequently, the two fluids exit in the *opposite* directions from which they entered: Outlet 1 contains blue fluid and Outlet 2 contains yellow fluid. When the chip is oriented at −90° relative to gravity **(C)**, the fluids rotate 90° clockwise to orient the more-dense blue fluid on the bottom of the channel and the less-dense yellow fluid on the top. When the channel splits into two outlets, each outlet receives an identical mixture containing 50% yellow fluid and 50% blue fluid. Finally, when the chip is oriented at 90° **(D)**, the fluids rotate 90° *counterclockwise* to once more orient the more-dense blue fluid on the bottom of the channel, and again both outlets contain identical mixtures containing 50% yellow and 50% blue. In this manner, the orientation of a microfluidic chip may be used to route fluids in different directions on-chip without using any off-chip control hardware.

**Fig 3:**
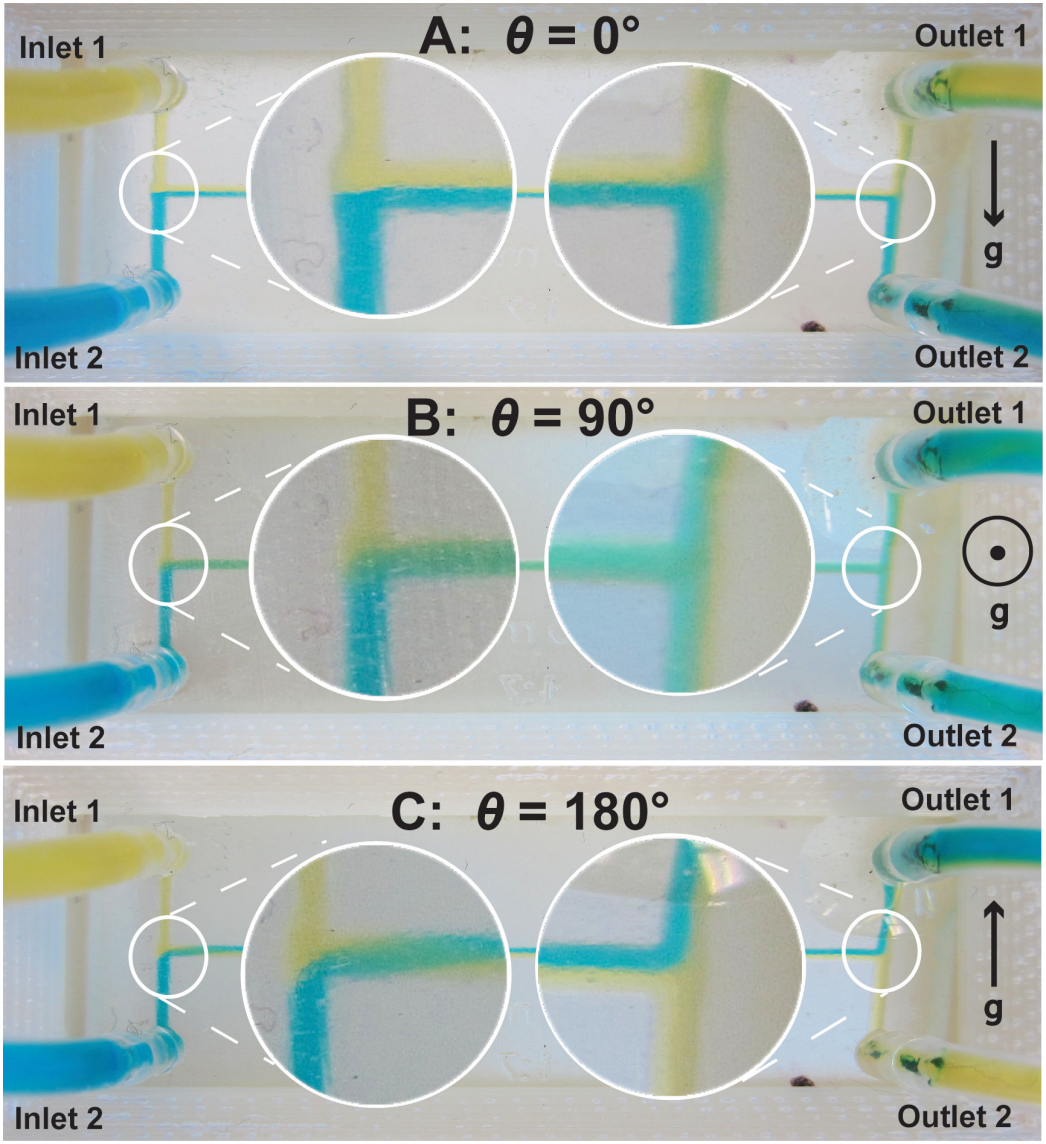
Photographs of a microfluidic mixer chip oriented at different angles *θ* relative to gravity. In each case Inlet 1 contains a less-dense yellow fluid (water; density *ρ* = 1.00 g/mL) and Inlet 2 contains a more-dense blue fluid (sucrose solution; *ρ* = 1.07 g/mL). When *θ* = 0° **(A)** the arrangement of yellow and blue fluids in the horizontal channel remains unchanged, and Outlet 1 contains yellow fluid and Outlet 2 contains blue fluid. However, at *θ* = 90° **(B)** gravity causes the more-dense blue fluid to move to the bottom of the horizontal channel and the less-dense yellow fluid to move to the top. This twists the contents of the horizontal channel by 90° and causes both outlets to contain identical mixtures containing ∼50% blue and ∼50% yellow. Finally, at *θ* = 180° **(C)** the gravity-induced repositioning of the fluids in the horizontal channel causes the fluids to twist by 180°, effectively swapping places in the channel. As a result, the two fluids exit the chip in directions *opposite* from where they entered, with Outlet 1 containing blue fluid and Outlet 2 containing yellow fluid.

To explore the effects of chip orientation on fluid flow inside a microfluidic chip, we used finite element analysis software to simulate the behavior of a simple microfluidic chip held at different angles relative to gravity. Our model combines the Navier-Stokes equations, Fick’s law of diffusion, and a function that describes the density of a solution as a function of the concentration of a solute [12]. COMSOL Multiphysics (Burlington, MA) was used to simulate a orientation-controlled mixer chip shown in Fig 2. The model simulated the experimental conditions in Fig 3: a microfluidic channel with a circular cross section and 1.0 mm diameter, a less-dense yellow fluid with density 1.00 g/mL, and a more-dense blue fluid with density 1.07 g/mL. The fluid phase in the model is governed by the continuity equation for the incompressible Navier-Stokes equations:

$$\begin{array}{c} \nabla \cdot u=0 \end{array}$$ (1)

$$\begin{array}{c} \rho u\cdot \nabla u=-\nabla P+\rho g+\nabla \cdot (\mu \left(\nabla u+\nabla u^{T}\right)) \end{array}$$ (2)

where *u* is the velocity vector, *μ* is the fluid viscosity, *P* is the pressure applied to the upstream end of the fluid, *ρ* is the density of the fluid, and *g* is the gravitational acceleration of objects on Earth (9.8 m s^−2^). The solute concentration follows the equation of conservation of mass and Fick’s Law of Diffusion:

$$\begin{array}{c} u\cdot \nabla c=\nabla \cdot (D\nabla c) \end{array}$$ (3)

where *D* is the diffusivity of the solute and *c* is the concentration of the solute. Eqs 1–3 are coupled through the dependence of a solution’s density on its solute concentration, which can be expressed as

$$\begin{array}{c} \rho =\rho_{\text{water}}\left(1+Bc\right) \end{array}$$ (4)

where *ρ* is the density of the solution, *ρ*_water_ is the density of pure water (1.00 g/mL), *c* is the solute concentration, and *B* is an experimentally-obtained, solute-specific constant that correlates a solution’s density with its solute concentration (in this study, *B* = 127 L/mol for sucrose). Eqs 1–4 are solved for a steady state flow with inlet solute concentrations of 0 mol/L in Inlet 1 and 1 mol/L in Inlet 2.

The H-shaped microfluidic channel network simulated in Fig 2 has two inlets and two outlets. Inlet 1 contains a yellow fluid with density *ρ* = 1.00 g/mL, and Inlet 2 contains a slightly-more-dense blue fluid (*ρ* = 1.07 g/mL). In Fig 2A, the chip is oriented such that Inlet 2 is in the same direction as gravity (so the angle *θ* between Inlet 2 and gravity is 0°). In this orientation, the force of gravity keeps the more-dense blue fluid flowing along the bottom of the channel and the less-dense yellow fluid flowing along the top of the channel. Consequently, when the channel splits into two outlets, the fluids leave in the same directions they came from: the more-dense blue fluid exits through Outlet 2 in the direction of gravity, and the less-dense yellow fluid exits through Outlet 1.

In Fig 2B, the chip has been rotated about the middle channel axis by 180°. In this configuration, the force of gravity causes the two fluids to quickly swap places in the middle channel so that the more-dense fluid flows along the bottom of the channel and the less-dense fluid flows along the top. When the channel splits, the fluids exit in the *opposite* direction they came from: the more-dense blue fluid entered from Inlet 2 but exits through Outlet 1, and the less-dense yellow fluid entered from Inlet 1 but exits through Outlet 2. In this manner two different fluids can be routed to two different destinations on-chip by orienting the chip at either 0° relative to gravity (Fig 2A) or 180° (Fig 2B).

In Fig 2C, the chip has been rotated by 90° relative to gravity. The force of gravity again causes the fluids to reorient to place the more-dense blue fluid on the bottom and the less-dense yellow fluid on the top (a clockwise rotation of 90°). When the channel splits, both Outlet 1 and Outlet 2 have the same contents: the bottom-half of each channel is filled with more-dense blue fluid, and the top half of each channel is filled with less-dense yellow fluid. After the contents of each exit channel becomes homogeneous by *e.g*. diffusional mixing, both outlets contain identical mixtures consisting of 50% blue and 50% yellow.

Finally, in Fig 2D, the chip has been rotated by −90° relative to gravity. This case is similar to Fig 2C; gravity once more causes the two fluids to reorient to place the more-dense blue fluid on the bottom and the less-dense yellow fluid on top (a *counterclockwise* rotation in this case) and the exit channels each contain the same mixture (50% yellow and 50% blue).

The results in Fig 2 can be generalized for any angle of orientation *θ*. If the less-dense fluid at Inlet 1 contains a solute *A* with a concentration [*A*_0_], and the more-dense fluid at Inlet 2 contains a solute *B* with concentration [*B*_0_], the concentrations [*A*] and [*B*] in each of the outlet channels are:

$$\begin{array}{c} \begin{array}{ccccc} \left[A\right] & =\left[A_{0}\right]\frac{\theta }{180} & \left[B\right] & =\left[B_{0}\right]\left(1-\frac{\theta }{180}\right) & \text{at}\;\text{Outlet}\;\text{1}\\ \left[A\right] & =\left[A_{0}\right]\left(1-\frac{\theta }{180}\right) & \left[B\right] & =\left[B_{0}\right]\frac{\theta }{180} & \text{at}\;\text{Outlet}\;\text{2} \end{array} \end{array}$$ (5)

Using the above equations, we can predict the concentrations [A] and [B] flowing in a chip held at any desired angle *θ*. These equations only apply to a microfluidic chip with circular cross-section channels, although similar equations may be derived for other channel shapes.

## Materials and Methods

To demonstrate the principle of orientation-based control of fluid flow in a microfluidic chip, we fabricated microfluidic chips similar to the one simulated in Fig 2. Microfluidic chips were designed using SolidWorks (Dassault Systèmes, Vélizy-Villacoublay, France), exported as an.STL file, and printed using a 3D printer (Form 1+, Formlabs, Cambridge, MA). These chips contain a H-shaped microchannel 42 mm long and 1 mm in diameter. The Stereolithography-based printer uses a 405 nm Class 1 laser to polymerize a liquid resin into a solid part. The resin used in this study is a combination of methacrylated monomers and oligomers.

Fig 3 shows our test chip in operation. Inlet 1 contains water, density *ρ* = 1.00 g/mL, and Inlet 2 contains more-dense sucrose solution, *ρ* = 1.07 g/mL. Sucrose solutions of precisely known densities were prepared using our software NaCl.py (available for download at http://groverlab.org). FD&C Blue #1 and Yellow #6 were used specifically for visual characterizations. The inlets were connected by tubing to fluid reservoirs that were held 3 cm above the chip. Since the inlet reservoirs were higher than the outlet reservoirs, a head pressure *P* = *ρgh* developed that pumped fluid flow from the input reservoirs through the chip and to the output reservoirs (*g* = 9.8 m s^−2^ and *h* = height difference between input and output reservoirs). The inlet and outlet reservoirs were maintained at the same height regardless of the tilt of the chip. Consequently, the orientation of the chip does not affect the flow rate. No off-chip fluid control hardware like pumps or valves were used.

The phenomenon of orientation-based control depends upon convection, not diffusion, being dominant in the microfluidic system. A dimensionless parameter called the Peclet number, *Pe*, is used to determine whether convection or diffusion is dominant in a system:

$$\begin{array}{c} Pe=\frac{Lu}{D} \end{array}$$ (6)

where *L* is the characteristic length, *u* is the flow velocity, and *D* is the solute’s diffusion coefficient. The calculated Peclet number for our flow rate (∼11,000) is much greater than one, indicating that convection is dominant and diffusional mixing between the different fluid streams is negligible.

Chips of different channel geometries were held at different angles. The average residence time of fluid in the chip was calculated to be 0.7 seconds. To make sure that the system reaches steady state, samples were collected from the chip after 1 minute of flow. Samples of 5 mL were collected from each outlet for each orientation and chip geometry. To quantify the resulting mixing ratios, fluids from both outlets were collected and analyzed using an UV-Vis-NIR spectrophotometer (V-670, Jasco, Easton, MD).

## Experimental Results

When the chip is oriented on its edge relative to gravity in Fig 3A (*θ* = 0°), the more-dense blue fluid remains on the bottom of the horizontal channel and the less-dense yellow fluid remains on the top of the channel. Consequently, the fluids exit the mixer chip in the same directions from which they entered (yellow fluid at Outlet 1 and blue fluid at Outlet 2). This case is identical to the simulation shown in Fig 1A. When the chip is held flat relative to gravity in Fig 3B (*θ* = 90°), the two fluids reorient relative to gravity (rotating 90° clockwise) to place the more-dense blue fluid at the bottom of the horizontal channel and the less-dense yellow fluid on the top of the channel. When the horizontal channel splits, both output channels have the same contents (∼50% yellow and ∼50% blue, which appears as green in the outlets). This situation is identical to the simulation shown in Fig 2C. Finally, when the chip is oriented on its *other* edge in Fig 3C (*θ* = 180°), the yellow and blue fluids swap places in the horizontal channel to place the more-dense blue fluid on the bottom and the less-dense yellow fluid on the top. Consequently, the fluids leave the Outlet channels *opposite* from where they entered, with yellow fluid at Outlet 2 and blue fluid at Outlet 1.

**Fig 4:**
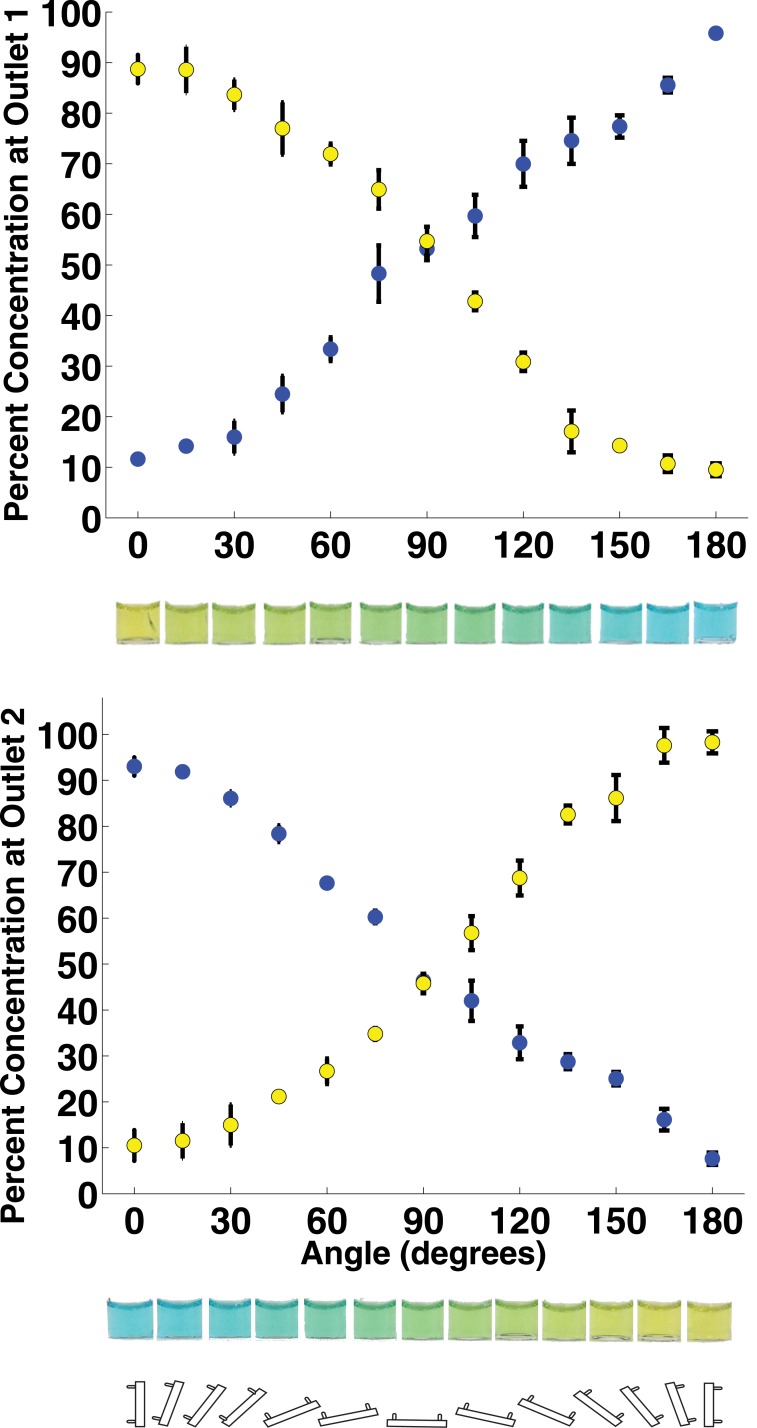
Concentrations of yellow and blue dyes in the mixer chip’s two outlets as the angle of rotation of the mixer chip is varied from 0° to 180°. Also shown are photographs of the fluid collected at each outlet and an illustration of the chip’s orientation at each angle of rotation. *N* = 3 measurements for each point; error bars indicate ±1 standard deviation. These results show that mixture composition is a function of the angle of rotation of the chip, and any desired mixture can be generated simply by orienting the chip and the required angle.

To demonstrate orientation-based control of fluid flow over a wide range of angles (not just the three angles shown in Fig 3), the mixer chip was operated at angles from 0° to 180° in 15° increments. As before, the more-dense blue fluid was a sucrose solution (density = 1.07 g/mL) and the less-dense yellow fluid was water (density = 1.00 g/mL). Fig 4 shows the concentration of each dye in the two output channels. As the mixer chip’s angle of rotation *θ* is varied from 0° to 180°, the concentration of blue fluid rises and yellow fluid drops in Outlet 1, and an opposite trend is observed in Outlet 2. The relationship of concentration on angle of rotation is roughly linear as predicted by Eq 5. This experimental data deviates from the predicted model at angles near 0° and 180°, where the outlet fluids concentrations are not 100% and 0% as predicted but ∼90% and ∼10% instead. This is likely due to diffusion within the horizontal channel in the mixer chip, which contributes a small amount of mixing between the two fluid streams. The effect of this diffusional mixing is most pronounced at angles near 0° and 180° where the outlets should contain pure (unmixed) fluids according to Eq 5; instead their contents are ∼90% pure. Additionally, the relationship of fluid concentration on angle of rotation in Fig 4 is not purely linear but appears to have some higher-order shape. This can be attributed to the cross-sectional geometry of the 3D-printed microfluidic channel because of the limited resolution of the 3D printer. The 1 mm channel was printed using a stereolithography 3D printer with a tolerance of ± 200 *μ*m. This could result in a microfluidic channel that is not perfectly circular and requires a more complex model than the one shown in Eq 5. However, the error bars in Fig 4 confirm that the outlet concentrations are a reproducible and predictable function of the angle of rotation of the chip, and a higher-order function could easily be derived that predicts the fluid concentrations in a chip at any rotation angle *θ* to within a few percentage points.

**Fig 5:**
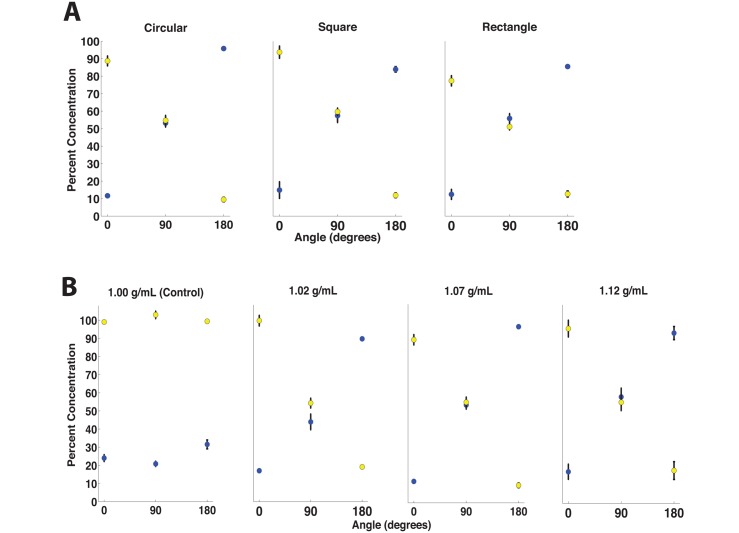
**(A)** Concentrations of yellow and blue dyes at Outlet 1 in mixer chips held at 0°, 90° and 180°, for chips with circular, square, and rectangular channel cross-sections. Chip orientation can be used to control on-chip fluids in devices with a variety of channel cross-sectional shapes, although performance deteriorates somewhat in the rectangular channel. These results suggest that microfluidic channels with aspect ratios close to one are best suited for orientation-based control. **(B)** Outlet fluid concentrations measured at Outlet 1 while orienting the mixer chip at 0°, 90° and 180° for four different fluid densities at Inlet 1. The density of fluid at Inlet 2 was kept constant at 1.00 g/mL. When there is no difference in fluid densities between the two inlet fluids, fluid reorientation is not observed and orientation-based control cannot be used (Control case). However, if the fluid density in Inlet 1 is just 2% greater than the fluid density in Inlet 2, then the flowing fluids reorient with respect to gravity and orientation-based control is possible.

To explore the role of channel cross-section shape in orientation-controlled microfluidics, we designed and 3D printed mixer chips with square (1 × 1 mm) and rectangular (1 × 1.25 mm) cross-section channels. We then repeated the experiments from Fig 5A using the square and rectangular chips for rotation angles *θ* = 0°, 90°, and 180°. The results in Fig 5A confirm that the orientation of a chip can still be used to control fluid mixing in chips with square and rectangular cross sections, though increased deviation from the predicted fluid concentrations is observed in the rectangular cross-section chip. This suggests that orientation-based control of microfluidics works best in channels with aspect ratios near one; at higher aspect ratios the channel geometry hinders the desired rotation of fluid in the channel. We were limited to a 1 mm channel diameter since we wanted to explore different geometries and aspect ratios using a 3D printer; however, the same phenomena were observed in a conventional glass microfluidic channel of 180 *μ*m (data not shown). We also examined the behavior of two fluids of different densities flowing in rectangular channels with much higher aspect ratios (cross-sectional dimensions 1 mm × 5 mm; data not shown). The experimental results further support the assertion that orientation-based control of microfluidics is most practical in channels with cross-sectional aspect ratios near one (circular and square channels).

In addition to channel geometry, fluid density has an effect on our phenomenon. The speed at which rotation occurs is influenced by the density difference. However, in practice we found that all rotations we observed were nearly instantaneous. The channel length was kept excessively long for characterization purposes. However, for a channel diameter of 1 mm, a minimum length of 10 mm is required for the rotation to occur. The larger the difference between fluid densities, the quicker the rotation occurs. Rotation speed could be increased by further increasing the difference between fluid densities; however, the resulting increase in concentration gradients will result in an increase in diffusive flux, potentially allowing for more mixing between the two layers.

To determine how much of a difference in density between the two fluids is necessary to employ orientation-based control, sucrose solutions with different concentrations (and thus different densities) were prepared as shown in Fig 5B. Experiments were performed using solutions with densities of 1.00 g/mL (control), 1.02 g/mL, 1.07 g/mL, and 1.12 g/mL. As before, the density of the second fluid was kept constant at 1.00 g/mL (water). In the control case in Fig 5B, there is no difference in densities between the two fluids in the mixer chip, so no reorientation of the fluids occur within the chip and the fluid concentrations at the outlets are constant regardless of the angle of orientation of the chip; however, when the density of one input fluid is increased by only 2% to 1.02 g/mL, the fluids again reorient with respect to gravity and orientation-based control is demonstrated.

## Conclusions

We demonstrated that the orientation of a microfluidic chip can be used to precisely control the flow of fluids inside the chip. By using the orientation of a chip to control fluid flow instead of on-chip valves or off-chip pumps and regulators, our technique can eliminate a substantial portion of the cost, size, and power-consumption of a microfluidic assay or instrument. Thus, this technique should facilitate the spread of microfluidic technologies to new applications in resource-limited and point-of-care settings.

Orientation-based control of microfluidics does depend upon different fluids in the chip having different densities. However, the amount of density difference necessary to use orientation-based control is very small (only ∼2%), and there are many different substances that can be added to a fluid to adjust its density. For applications that do not require precise control of the osmotic strength of fluids, small amounts of solutes like sucrose (as used here) and sodium chloride can be easily and inexpensively added to a fluid to enable orientation-based control. For applications in which the osmotic strength of the fluid *does* need to be controlled, a number of compounds have been developed that increase a fluid’s density without affecting its osmotic strength. These include Ficoll, a high molecular weight polysaccharide used in density gradient centrifugation [13]; Percoll, a colloidal silica solution which is biologically inert and also used in density gradient centrifugation [14]; Visipaque (iodixanol), an isotonic, nonionic, nontoxic compound used as an intravenous contrast agent in radiography [15] and metatungstate solutions, a dense, inert and inorganic solutions with low reactivity. By adding small amounts of substances like these to fluids and using the principle of orientation-based control, microfluidic assays can run with little or no off-chip hardware.

A density difference of ∼2% is required for fluid reorientation to occur. Increasing the density of a solution by ∼2% may change the viscosity of the solution. For example, increasing the density of a sucrose solution by 2% increases the effective viscosity of the solution by 17%. However, this viscosity difference is actually too low to affect the concentration of the output fluid. Karst *et al*. [12] studied fluids of different viscosities flowing alongside each other in laminar flow (as is the case in the horizontal channel of our chip). They found that the ratio of the volumes occupied by each fluid inside the tube is not equal to the ratio of the fluids’ flow rates. Moreover, they found that the viscosity of the second fluid has to be at least 100% greater than the viscosity of the first fluid for the ratio of the volumes of the two fluids in the channel to be altered. Therefore, the effect of fluid viscosity is minimal.

Finally, is orientation-based control powerful enough to control real-world microfluidic chips? Certainly chips with hundreds of computer-controlled valves offer a level of fluid control that may be unattainable by orientation-based control (though this level of control comes with a significant cost). However, many real-world microfluidic devices require simpler fluid control and could be controlled using chip orientation. For example, the proof-of-concept mixer chip shown here could be used in a drug toxicity screening assay by exposing cells downstream of the mixer to various concentrations of a drug [16]. This could be accomplished by flowing a drug solution in one inlet and a diluent with 2% greater density in the second inlet, and trapping cells in one of the outlet channels. By orienting the chip at a certain angle and then assessing the viability of the cells, the response of the cells to a particular concentration of the drug could be assessed. Assuming that a chip can be held at an angle with an accuracy of ±5 degrees, the resulting drug concentration should be accurate to about ±10% which is adequate precision for many drug screening asays. This is one of many real-world microfluidic assays and diagnostics that could be performed with little or no off-chip control hardware using orientation-based control.
